# Supplementary material for: An improved nucleic acid sequence-based amplification method mediated by T4 gene 32 protein
Source: PLoS One. 2022 Mar 24;17(3):e0265391. doi: 10.1371/journal.pone.0265391 (PMC8947125; doi:10.1371/journal.pone.0265391)
Supplement: S1 File — (DOCX) [file pone.0265391.s001.docx]

Supporting Information for

**An Improved Nucleic Acid Sequence-Based Amplification Method Mediated By T4 Gene 32 Protein**

Yi Heng Nai^*^, Egan H. Doeven & Rosanne M. Guijt*

*^1^ Centre for Regional and Rural Futures, Deakin University, Geelong, Victoria 3220, Australia*

*Co-corresponding Authors:
Yi Heng Nai, Ph.D. Email: [ryan.nai@gmail.com](mailto:ryan.nai@gmail.com) ;

Prof. Rosanne M. Guijt, Email: rosanne.guijt@deakin.edu.au

**Supplementary Table 1.** Sequences of the NASBA primers and its corresponding-specific molecular beacons, with each molecular beacon labeled with 6-Carboxyfluorescein (6FAM) fluorophore at 5’ end-and Blackhole quencher-1 (BHQ-1) at 3’ end. The quantitation of synthetic RNA was performed using real-time PCR analysis using the Gag primers for HIV-1 assay [1].

| Name | Method | Sequence (5'-3') |
| --- | --- | --- |
| HIV-1 P1 | NASBA | *AATTCTAATACGACTCACTATAGGG*TGCTATGTCACTTCCCCTTGGTTCTCTCA |
| HIV-1 P2 | NASBA | AGTGGGGGGACATCAAGCAGCCATGCAAA |
| Molecular Beacon | NASBA | F-GCATGCATCAATGAGGAAGCTGCAGAATGGGAGCATGC -Q |
| HIV-1 gag gene sequence (Accession #k03455 nt 1359-1499) | VLP | TGAAGGGTACTAGTAGTTCCTGCTATGTCACTTCCCCTTGGTTCTCTCATCTGGCCTGGTGCAATAGGCCCTGCATGCACTGGATGCACTCTATCCCATTCTGCAGCTTCCTCATTGATGGTCTCTTTTAACATTTGCATGGCTGCTTGATGTCCCCCCACTGTGTTTAGCATGGTGTTTAAATC |
|  |  |  |
| NL1359F | RT-PCR | AGTGGGGGGACATCAAGCAGCCATGCAAAT |
| NL1500R | RT-PCR | TGCTATGTCAGTTCCCCTTGGTTCTCT |
| Taqman Probe | RT-PCR | FAM-ATCAATGAGGAAGCTGCAGAATGGGA-BHQ-1 |

*** In italic font is the T7-promoter recognition sequence depicted; underlined are the nucleotides that form the stem of the typical stem-and-loop structure of the molecular beacon*

**Quantitation of VLP extracted HIV-1 gag RNA by qRT-PCR**

The purified RNA was serially diluted linearly by 10-fold in RNase-Free Tris EDTA (pH 7.5) buffer, and the quantitation of the series was perform using qRT-PCR using hydrolysis probe method (Bioline SensiFAST™ Probe No-ROX One-Step Kit, Bioline AUS, Sydney) with HIV-1 gag primer-probe set (Table S1). Following figures showed the serially diluted RNA samples and its corresponding Ct values. the calibrated nucleic acid extracts were transferred to an 8-well strip of 0.2 ml polypropylene tubes at stored under -80 °C until required.


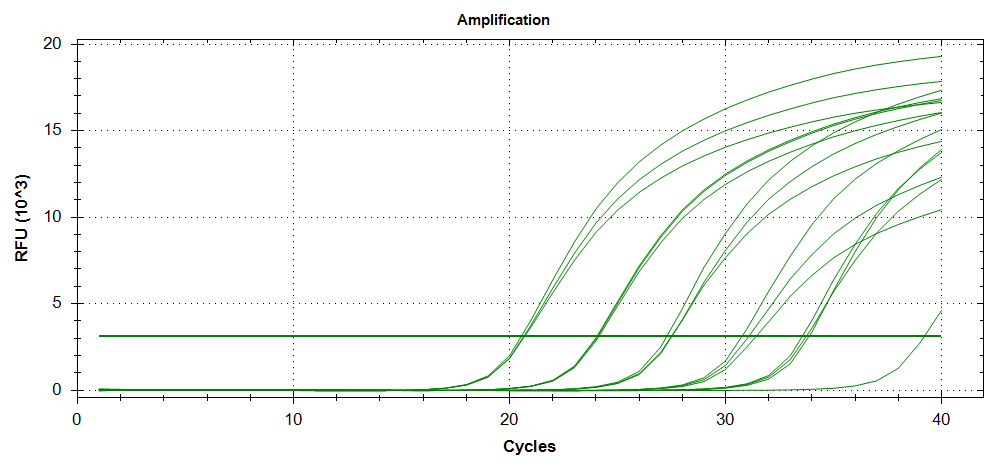


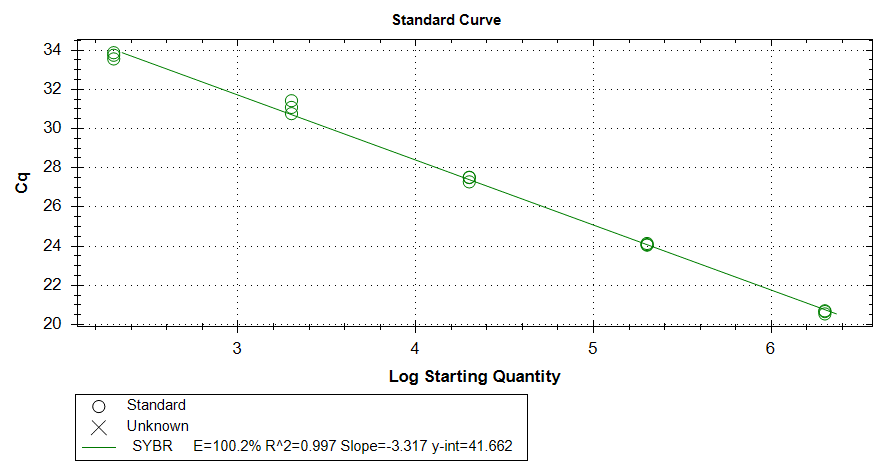


- **Effect of various concentration gp32 on NASBA - Full length HIV-1**

**
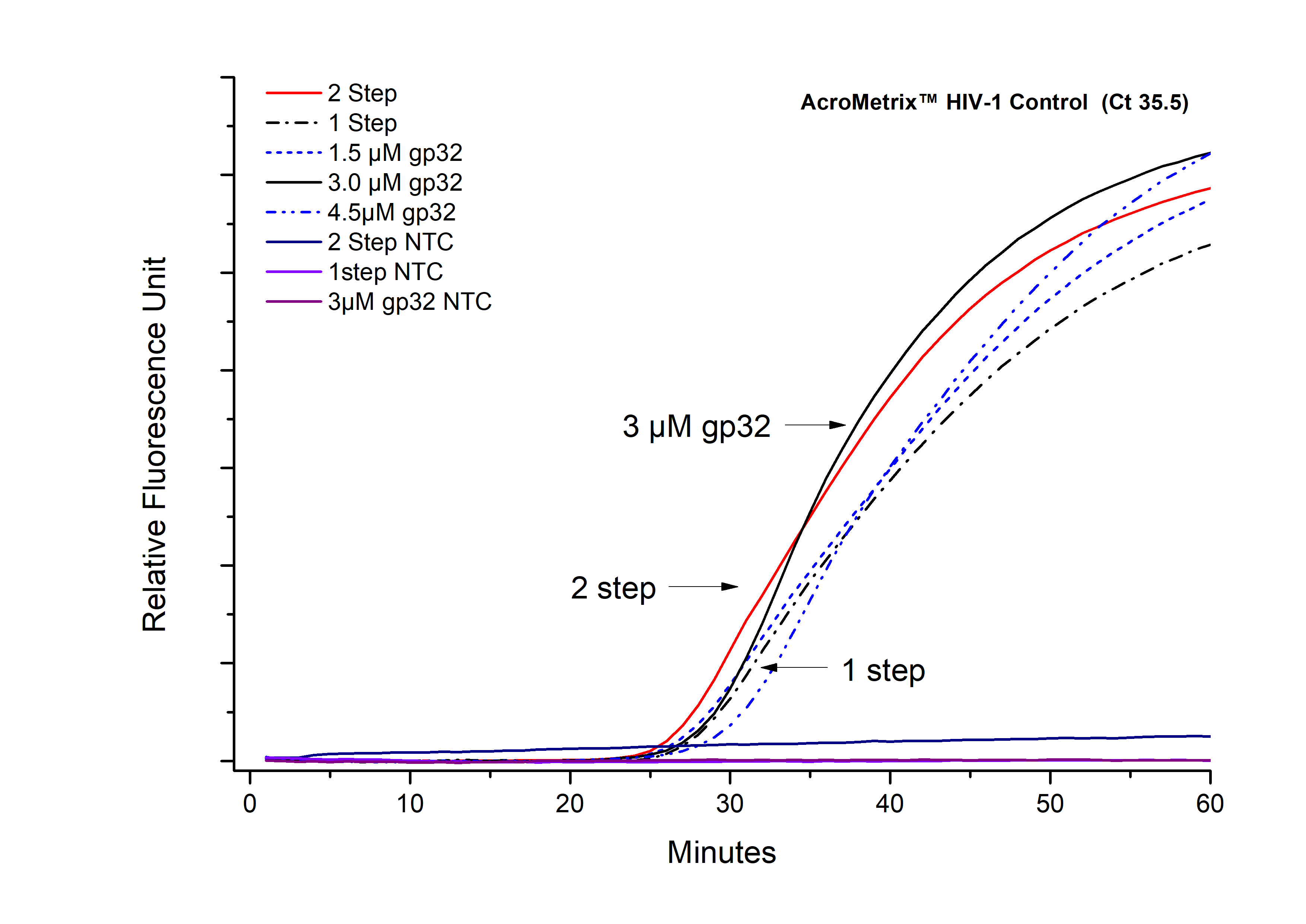
**

**References**

1. Ohishi M, Nakano T, Sakuragi S, Shioda T, Sano K, Sakuragi J-i. The relationship between HIV-1 genome RNA dimerization, virion maturation and infectivity. Nucleic acids research. 2011;39(8):3404-17.
