# Supplementary material for: An improved nucleic acid sequence-based amplification method mediated by T4 gene 32 protein
Source: PLoS One. 2022 Mar 24;17(3):e0265391. doi: 10.1371/journal.pone.0265391 (PMC8947125; doi:10.1371/journal.pone.0265391)
Supplement: Attachment — Submitted filename: Review comments (PONE-D-21-08924).docx [file pone.0265391.s002.docx]

**Review Manuscript**

Title: An Improved Nucleic Acid Based Sequence Amplification Method Mediated by T4 Gene 32 Protein

Manuscript ID: PONE-D-21-08924

Authors: Yi Heng Nai^*^, Egan H. Doeven, and Rosanne M. Guijt

Submitted to PLOS ONE

This manuscript describes an improved nucleic acid sequence-based amplification (NASBA) using single-stranded binding protein (SSB), T4 gene protein 32 (T4gp32). In this strategy, three SSBs were employed to construct a modified NASBA capable of eliminating the initial denaturation step and thus amplifying target RNA molecule in a single pot at 41 ℃. The authors applied this strategy to amplify synthetic HIV-1 RNA molecules. The initial denaturation step in the traditional NASBA is needed to disrupt the complicated secondary structure of long genomic RNAs and the authors should validate the benefits of this improved NASBA technology by amplifying long genomic RNAs but not short synthetic RNAs which might rarely require the initial denaturation. Furthermore, this manuscript is not well-structured and contains a lot of errors. Therefore, I would not recommend acceptance of this manuscript. Some of my other comments are as follows.

Comment 1. Nucleic Acid Based Sequence Amplification’ should be corrected to ‘Nucleic Acid Sequence-Based Amplification (NASBA)’ throughout the overall manuscript.

Comment 2. Many abbreviations such as ttp and ET SSB are not defined.

Comment 3. The authors are recommended to provide standard deviation for Ct data in Figure 2.

Comment 4. The authors need to present the experimental data obtained from RT-qPCR conducted to confirm the concentrations of target RNA.

Comment 5. The authors need to provide relevant references

1) Related to false positives of the NASBA reaction. (Page3, Line 59)
2) Related to the thermolability of the T7 RNA polymerase. (Page 4, Line 65)
3) Related to the T4gp32 effects including the increase of the DNA sequencing read length and alleviation of the PCR inhibition. (Page 5, Line 106)
