# Supplementary material for: An improved nucleic acid sequence-based amplification method mediated by T4 gene 32 protein
Source: PLoS One. 2022 Mar 24;17(3):e0265391. doi: 10.1371/journal.pone.0265391 (PMC8947125; doi:10.1371/journal.pone.0265391)
Supplement: Attachment — Submitted filename: Response to reviewers (PONE-D-21-08924).docx [file pone.0265391.s003.docx]

**Review Manuscript**

Title: An Improved Nucleic Acid Based Sequence Amplification Method Mediated by T4 Gene 32 Protein.

Manuscript ID: PONE-D-21-08924

Authors: Yi Heng Nai^*^, Egan H. Doeven, and Rosanne M. Guijt

Submitted to PLOS ONE

**We would like to thank the editor and reviewers for their patience. The lengthy revision process has been due to COVID-19 outbreak lockdown (82 days since late June) in Victoria Australia, excluding us from the laboratory for extended periods of time, as well as impacting on the supply chain. For example, we waited from mid-May to early September for the AcroMetrix™ HIV-1 Controls to arrive from sole supplier Thermo Fisher Scientific (AUS).**

*Reviewer #1: The manuscript from Nai, Doeven and Guijt describes an improvement to NASBA reactions by inclusion of single-stranded binding protein, particularly T4 gp32. This would be a good benefit to improving usability and application of NASBA, but I have a few points that I think should be addressed before acceptance for publication.*

*1) As the authors describe, an initial denaturation step at 60-65C is typically done before adding NASBA enzymes and running the detection reaction. But the reason for this step is pretty fundamental, typically NASBA reactions do not work at all without it. Here the data show that the "one-step" protocol is actually just fine, a few minutes slower than the standard two-step but otherwise amplifying and detecting target well. This is an unusual result and I wonder if the simplicity of the used template or something else about the particular reaction being done here gives rise to an abnormal finding. If the key bottleneck for wider use of NASBA is this heat step, it seems from the data here that that step is already unnecessary. It would be good to investigate other primers/targets to demonstrate that the gp32 effect is not assay-specific, but it could also add to this point which is very unexpected to me.*

Response: We thank Reviewer #1 for the comment and questions. We acknowledge that the simplicity of synthetic template may played a part in this interesting observation described in the manuscript, and have endeavoured to investigate the effect of gp32 with full length genomic RNA sample (Thermo Scientific™ AcroMetrix™ HIV-1 Mid Control). HIV-1 was selected for the characterization of the SSBs in NASBA because HIV-1 is one of routine diagnostic assays performed at a commercial scale on bioMérieux (NASBA) platform, and as Reviewer 1 mentioned, the Hologic Panther (TMA) platform.

The full length HIV-1 control has a Ct value of 35.5 which translate to sub 100 copies per reaction (5000 copies/mL, 50 µL elution, 1 µL purified RNA into RT-PCR/NASBA). This is a very dilute sample, but higher concentration HIV-1 samples were inaccessible to the authors due to rigorous institutional infectious disease control policies and procedures. gp32 was still able to provide improvement in TTP compared to 1 step NASBA, albeit the effect was small (see attached and supplementary); ttp decreased from 29 to 28.5 min. Based on non-disclosed results obtained using Avian influenza A H5N1 RNA included below (NASBA Adapted D148/D149 H5 /IVAH5 MB primer set) under similar conditions, 3 µM gp32 improved ttp from 38 to 31 min, demonstrating the efficacy of gp32 for longer RNA target.

From personal communication with our collaborators/reagent suppliers at Life Sciences Advanced Technologies, we learned that there are significant differences in the need for the thermal annealing between primer sets. Based on this and our experimental results presented in the manuscript and provided above for AIV H5N1 RNA, we respectfully disagree with reviewer#1*“This is an unusual result and I wonder if the simplicity of the used template or something else about the particular reaction being done here gives rise to an abnormal finding.”*

While we cannot experimentally exclude the target concentration as a cause for the limited improvement in ttp for the HIV-1 samples, we have experimentally demonstrated a minor improvement in ttp using this primer set that provides a surprisingly fast ttp in absence of gp32 and thermal annealing. The noticeable improvements for the shorter VLP target as well as for AIV H5N1 extract – both primer sets where significant delays are observed when eliminating the thermal annealing step - strongly suggest a role of SSB in NASBA (or TMA), demonstrating the feasibility for elimination of the thermal annealing step in NASBA, and presenting an opportunity for the elimination of an important disadvantage of this RNA specific isothermal amplification method. We trust that communicating this finding will trigger further studies to unravel the multifaceted role(s) gp32 plays in NASBA, as eluted to in the manuscript.

*2) The authors describe better usability of NASBA for point-of-care settings, but the reaction requires a molecular beacon and real-time fluorescence as well as extracted RNA. Could the detection be done more simply?*

Response: The purpose of this manuscript is to demonstrate a SSB to eliminate the need for a thermal annealing step and to make NASBA more attractive for point-of care settings. Because of the fundamental nature of eliminating a processing step, we employed the most commonly used detection approach using a molecular beacon and real-time fluorescence. While we expect full compatibility with alternative detection approaches that may be more com partible with in-field use, those investigations are out of scope of the current communication.

*Could the VLPs be used directly without extracting RNA? I also don't see the benefit of encapsulating a synthetic RNA then extracting it again, if a mock sample is desired here then a real mock sample should be used, spiking the VLPs into a swab or blood or whatever.*

Response: No. We believe VLP cannot be used directly without extraction, as the RNA would be still remain within theVLP capsid at non-denaturing NASBA reaction buffer condition. Besides, the VLP was used to provide calibrated inputs during our investigation because - compared to naked RNA - the VLPs tolerate the exposure to RNase. We indeed obtained them for spiking into complex, RNase containing matrices like blood and saliva at a later stage, but working in these matrices is beyond the scope of this contribution. Here, their purpose is limited to ensuring interday repeatability.

*3) Some statistical analysis would help to show that the benefits/differences between the conditions are significant. The authors at one point compare 17.3 and 18 minutes, for example, and the data in Figure 3 would benefit from some significance determination. In Figure 3 the error bars in (a) seem extremely small compared to (b), the number of replicates is not listed and I find the difference odd.*

Response: We thank reviewer #1 for identifying this oversight and now included the standard deviation in text (Page 5 line 102-103), with the data used for Figure 2 (n=3). We recognized that recognizing n=3 is insufficient for thorough statistical analysis, we found that the Ct value with gp32 (M=17.38, SD 0.018) was lower than without gp32 (M=18.00, SD=0.103), for a one tailed t test, t(2)=5.14, p=0.018.

For the experiments reported in Fig 3(a), the same RNA input was used, and a decrease in the standard deviation was noted for increasing gp32 concentration. SDs for all gp32 experiments were smaller than those obtained for the 2-step NASBA, but this may be due to the small sample size. For the experiments reported on in Fig 3(b), the SD increased with higher Ct value (e.g > Ct 34). For the Ct 20 and 23, comparable to Fig3(a), the SD for gp32 are 0.28 and 0.153, respectively. This is comparable with the 0.128 found for 3 µM gp32, indicated in Fig3(a). Moreover there is a break in the Y axis in Fig 3(b) , which may skewed the scaling between the error bars when compared to Fig 3(a)

*4) As the authors note there are other isothermal methods being more widely used, RPA, LAMP, etc. What's the benefit of NASBA over those? Is it just that NASBA is an alternative, or does it offer something else? Sensitivity shown here is pretty moderate and I'd think any of the methods could pick up 150 copies in 30 minutes, and some without requiring fluorescence detection. Some commentary on why NASBA would be useful, even if it's just "as COVID has demonstrated we need as many methods as possible".*

Response: Compared to other isothermal methods, NASBA is unique by directly amplifying RNA, providing selective amplification of RNA in the presence of genomic DNA. Specific detection of the amplification product can then be afforded by the use of molecular beacon hybridization strategy. As Reviewer #1 points out, newer isothermal methods such as LAMP and RPA have shown to be superior in speed and sensitivity and amenable for alternative detection strategies such as lateral flow assays for RPA and colorimetric detection for LAMP by NEB. However non-specific amplification remains an issue. RPA are known to be inherently susceptible to aberrant amplification <https://journals.plos.org/plosone/article?id=10.1371/journal.pone.0235216>, whereas the specific amplification in LAMP is an ongoing challenge and only recently mitigated by coupling additional specificity using CRISPR for detection [1, 2]. Colorimetric LAMP has a 30 min amplification cut off as longer amplification will result in color changes in the detection agent. This makes its application for the detection of low concentration samples highly dependent on primer set design and optimization. So while there is fierce competition between existing and upcoming amplification methods, each has its strengths and for NASBA it is the only approach directly targeting RNA.

*4) Minor points:
It would be good to show the molecular beacon binding the target in Figure 1. Also it's not very clear which strands are DNA and which are RNA as the images and labels are very small.*

Response: Thanks for this feedback, a revised version of Figure 1 is included in the revised manuscript.

*NASBA and TMA are of course not obscure, rarely-used methods but diagnostic workhorse methods performed by the many thousands or millions every day in platforms like the Hologic Panther. Would be good to note this, and that the 2-step temperature requirement isn't a problem for widespread use just for simple applications.
In the introduction the authors state that NASBA is "less prone to false positives and faster than alternatives relying on reverse transcription of the RNA into DNA." NASBA is only less prone to false positives with molecular beacon detection, certainly not if using an intercalating dye or other readout, and it of course requires reverse transcription of the RNA into DNA otherwise it doesn't work as the authors' own cartoon shows. This should be restated*

Response: We acknowledge the awkward wording in the manuscript, and revised the text as well as the illustrations in Figure 1 accordingly. The revised sentences now read

Line 60-64

NASBA is primarily employed to directly amplify RNA targets and **– using a molecular beacon for detection -** it was demonstrated to be less prone to false positives **in the presence of genomic DNA** [3, 4] and faster than alternatives relying on reverse transcription of the RNA into DNA **without the need for DNase I treatment.**

Additionally, the following section was added after line 68:

**Because T7 RNA polymerase is thermolabile, NASBA is executed as a 2-step process, adding the enzymes after the thermal primer annealing step[5]. Recognising NASBA and TMA are less frequently used than PCR, the assays are performed many thousands to millions times a day in platforms including bioMérieux Nuclesens^®^ and Hologic Panther^®^. Compared to other isothermal amplification methods, such as LAMP or RPA, the 2-step process could be considered inconvenient for high throughput or point of care operation as each step may present as an operational and engineering challenge [6]. Here, the potential of SSBs to facilitate primer binding and eliminate the thermal annealing step in NASBA is reported to make NASBA a single step assay.**

**Reviewer #2:**

*This manuscript describes an improved nucleic acid sequence-based amplification (NASBA) using single-stranded binding protein (SSB), T4 gene protein 32 (T4gp32). In this strategy, three SSBs were employed to construct a modified NASBA capable of eliminating the initial denaturation step and thus amplifying target RNA molecule in a single pot at 41 ℃. The authors applied this strategy to amplify synthetic HIV-1 RNA molecules. The initial denaturation step in the traditional NASBA is needed to disrupt the complicated secondary structure of long genomic RNAs and the authors should validate the benefits of this improved NASBA technology by amplifying long genomic RNAs but not short synthetic RNAs which might rarely require the initial denaturation. Furthermore, this manuscript is not well-structured and contains a lot of errors. Therefore, I would not recommend acceptance of this manuscript. Some of my other comments are as follows.*

Response: We would like to thank the reviewer for critically evaluating this manuscript. Despite our confidence in the presented results – validated by non-published data confirming gp32’s efficacy using avian influenza virus - additional experiments were conducted to confirm if the reported findings are applicable to long genomic RNA. Using full length HIV-1, we experimentally confirmed a nominal improvement in performance in presence of gp32, data supplied in the SI. The limited extent of the improvement is most likely due to the fact that the HIV-1 RNA control was at very low concentration, unfortunately we were unable to access higher concentration samples to investigate further. While it is clear that the mechanism of SSBs in primer annealing in NASBA (or TMA) needs further investigation and that optimization of reaction conditions is required for different primer sets, we have provided evidence that SSBs have potential to eliminate the thermal annealing step for a range of target lengths (HIV-1 gag geneVLP (430 nucleotides, Influenza HA gene 1765 nucleotides and HIV-1 9749 nucleotides)

*Comment 1. Nucleic Acid Based Sequence Amplification’ should be corrected to ‘Nucleic Acid Sequence-Based Amplification (NASBA)’ throughout the overall manuscript.*

Response: We thank the reviewer for highlighting omission of the hyphen, we rectified this throughout the manuscript, including the title.

*Comment 2. Many abbreviations such as ttp and ET SSB are not defined.*

Response: We thank the reviewer for the feedback and reviewed the manuscript for correct and consistent use of abbreviations. As a result, the revised manuscript define time-to-positivity (ttp) in the abstract, in addition to its definition at first mention in line 99.

Extreme Thermostable Single-stranded binding protein (ET SSB) Extreme Thermostable Single-stranded binding protein (ET SSB) and T4 gene gp32 protein (gp32) are now also defined in the abstact. For ET SSB we added the full name at first mention in line 87. We also removed the use of T4 gp32 on two occasions and gp32 is now used consistently. We ensured that with its introduction, transcription-mediated amplification (TMA) is defined.

*Comment 3. The authors are recommended to provide standard deviation for Ct data in Figure 2.*

Response: Error bars were added to the data, and the number of experiments per datapoint (n=3) was added top the caption to define the number of replicates.

*Comment 4. The authors need to present the experimental data obtained from RT-qPCR conducted to confirm the concentrations of target RNA.*

Response: We thank the reviewer for the comment. The data is now included in the supplementary information section.

*Comment 5. The authors need to provide relevant references*

We included the references below, listed by mention.

*Related to false positives of the NASBA reaction. (Page3, Line 59)*

Simpkins S, Chan A, Hays J, Öpping B, Cook N. An RNA transcription‐based amplification technique (NASBA) for the detection of viable Salmonella enterica. Letters in applied microbiology. 2000;30(1):75-9.

Cook N. The use of NASBA for the detection of microbial pathogens in food and environmental samples. Journal of Microbiological Methods. 2003;53(2):165-74.

*Related to the thermolability of the T7 RNA polymerase. (Page 4, Line 65)*

Boulain J-C, Dassa J, Mesta L, Savatier A, Costa N, Muller BH, et al. Mutants with higher stability and specific activity from a single thermosensitive variant of T7 RNA polymerase. Protein Engineering, Design & Selection. 2013;26(11):725-34.

*Related to the T4gp32 effects including the increase of the DNA sequencing read length and alleviation of the PCR inhibition. (Page 5, Line 106)*

Kreader CA. Relief of amplification inhibition in PCR with bovine serum albumin or T4 gene 32 protein. Applied and Environmental Microbiology. 1996;62(3):1102-6.

Rapley R. Enhancing PCR amplification and sequencing using DNA-binding proteins. Molecular biotechnology. 1994;2(3):295-8.

1.	Gootenberg JS, Abudayyeh OO, Kellner MJ, Joung J, Collins JJ, Zhang F. Multiplexed and portable nucleic acid detection platform with Cas13, Cas12a, and Csm6. 2018;360(6387):439-44. doi: 10.1126/science.aaq0179 %J Science.

2.	Joung J, Ladha A, Saito M, Kim N-G, Woolley AE, Segel M, et al. Detection of SARS-CoV-2 with SHERLOCK one-pot testing. New England Journal of Medicine. 2020;383(15):1492-4.

3.	Cook N. The use of NASBA for the detection of microbial pathogens in food and environmental samples. Journal of Microbiological Methods. 2003;53(2):165-74. doi: <https://doi.org/10.1016/S0167-7012(03)00022-8>.

4.	Simpkins S, Chan A, Hays J, Öpping B, Cook N. An RNA transcription‐based amplification technique (NASBA) for the detection of viable Salmonella enterica. Letters in applied microbiology. 2000;30(1):75-9.

5.	Boulain J-C, Dassa J, Mesta L, Savatier A, Costa N, Muller BH, et al. Mutants with higher stability and specific activity from a single thermosensitive variant of T7 RNA polymerase. Protein Engineering, Design & Selection. 2013;26(11):725-34.

6.	Maffert P, Reverchon S, Nasser W, Rozand C, Abaibou H. New nucleic acid testing devices to diagnose infectious diseases in resource-limited settings. European Journal of Clinical Microbiology & Infectious Diseases. 2017;36(10):1717-31.
