# Supplementary material for: An improved nucleic acid sequence-based amplification method mediated by T4 gene 32 protein
Source: PLoS One. 2022 Mar 24;17(3):e0265391. doi: 10.1371/journal.pone.0265391 (PMC8947125; doi:10.1371/journal.pone.0265391)
Supplement: Attachment — Submitted filename: Response R2.docx [file pone.0265391.s004.docx]

We would like to than the reviewers for their detailed comments and criticisms, which we addressed below. For trackability, the reviewers comments are pasted in black and aligned to the right, and our response and overview of changes made to the manuscript are indicated in blue and aligned to the left of the page.

Reviewer #1: I thank the authors for addressing the comments from the reviewers, the resulting updated version here is better but I still have a fundamental point that now if anything needs more attention. Also my intent from the first round was to get some of the points raised added into the manuscript, the authors seemed to disagree with me but I maintain that the things raised in the first review are still relevant, if no experiment is required just some discussion or text I fail to see how that is "out of scope of the current communication.

**Response**

We would like to apologise not having addressed detection in detail.

**Action taken:**

A sentence and reference on detection have now been included from line 92:

“A myriad of techniques has been developed for the detection and quantification of nucleic acid amplification products, ranges from simple colorimetric reactions for visual read-out to more complex electrochemical and optical sensors; a comprehensive review focusing on point of care analysis can be found elsewhere [18]. Here, we used the fluorescence signal from a molecular beacon for fluorescence detection.”

But more importantly, both I and reviewer #2 pointed out that the thermal denaturation/annealing step is generally considered required for NASBA/TMA to work well. The authors are claiming it is not when gp32 is present. That would be great, so in the assay shown matching the speed of the typical 2-step protocol just by including gp32 is a nice result...but it is very plainly a result of using this specific assay, as the 1-step, non-gp32 version works surprisingly well.

**Response:**

We agree that for HIV-1, the one step protocol worked surprisingly well, a phenomenon occasionally observed (but not reported) for NASBA amplifications. Unfortunately, we have been unable to identify “typical” attenuation for one step NASBA reactions for benchmarking purposes, as articles typically refer to the protocols developed in the 1990’s without presenting the attenuation experienced. We hypothesize it depends on the primers used as well as the formulation of the amplification buffer. A discussion of the observed amplification in single pot NASBA has now been included (see below under action taken).

The presented research shows that T4GP32 outperforms the other SSBs in specificity, not displaying non-specific amplification. We included a reference to recent review of approaches for enhancing isothermal amplification (Özay, B. and S.E. McCalla, A review of reaction enhancement strategies for isothermal nucleic acid amplification reactions. Sensors and Actuators Reports, 2021. 3: p. 100033). Please note this review confirms that SSBs so far have only been used to decrease non-specific binding, emphasizing the novelty of the use of gp32 in the primer annealing.

A possible explanation is that the reagents from LSAT may have been stabilised with trehalose -without our knowledge - which may have helped the one-pot reaction, as trehalose has been demonstrated to enhance isothermal amplification reactions (Mok, E., Wee, E., Wang, Y. *et al.* Comprehensive evaluation of molecular enhancers of the isothermal exponential amplification reaction. *Sci Rep* **6,** 37837 (2016). We have noted that the precise formulation is no longer provided by LSAT. Seven years ago was listed as 40 mM TrisHCl, pH 8.5 @ 25ºC, 12 mM MgCl2, 70 mM KCl, 5 mM DTT, 15% Dimethyl Sulfoxide ---- 2 mM rA,C,UTP, 1.5 mM rGTP, 0.5 mM Inosine 5'-triphosphate, 1 mM dNTP. LSAT has not mentioned a change in formulation, but simply removed the composition from the public domain. It is normal in the industry to keep the exact formulation of the buffer as a trade secret.

The performance enhancement by trehalose described in the Sci Rep article were obtained at concentrations of 0.4 M, a value that is unrealistic to be present as “contamination”, discrediting the hypothesis trehalose entered the buffer with the stabilised enzymes.

Importantly, we have experimentally tested most of the additives listed in the newly cited review [Özay, B. and S.E. McCalla)] to facilitate the single pot reaction, identifying gp32 as the most promising candidate for NASBA. In that study, the amplification performance in the LSAT NASBA buffer was similar to that in the buffer composed using the ingredients listed seven years ago, hence we have no experimental evidence the LSAT formulation may have changed (nor do we have evidence it did not). The study did confirm an enhancement in amplification for the single pot reaction in presence of trehalose, however, the gp32 results reported here were superior.

Considering we used the LSAT buffer as indicated in the method section of manuscript, the performance enhancement was found for gp32 but not for other SSBs, irrespective if we used the LST buffer or made our own, we continue to believe it is important to communicate our findings on gp32 to allow other researchers to elucidate and benefit from its action(s) in NASBA and other isothermal amplification methods.

**Action taken:**

Added to line 104/104

“Attenuated amplification was observed for all one step NASBA reactions.”

We also included

“ To the authors best knowledge, there are no literature reports of single step NASBA protocols, but anecdotal reports confirmed in our laboratory suggest the reliance on the thermal annealing step varies for primer/targets.”

Above this discussion to illustrate that the observed one-pot amplification is not unique, and has been observed, but not reported by others.

And After line 76, the following text was added, including the abovementioned reference

“A recent review on enhancement strategies for isothermal amplifications concluded that effective additives used to eliminate the thermal denaturation step include helicase, recombinases, endonucleases, ionic liquids, betaine, proline and trehalose [15], and SSBs used for decreasing non-specific interactions and to prevent nucleic acid degradation.”

Additionally, we replaced “eliminate” with “decrease the reliance on” to moderate the concluding statement.

And added

“ While mechanistic studies explaining the role of gp32 in one step NASBA, as well as to examine the effect of the target and primer are required, ..” to the conclusion

The authors own data in the rebuttal shows another amplicon where the 1-step gives very poor signal and the gp32 addition does not rescue it to the level of the 2-step, let alone faster as they (unconvincingly from the limited data points) claim in the manuscript. I feel this proves my point, not theirs, and the gp32/1-step effect may indeed by an artifact of the assay used in the main body of the text. 2 assays are shown here, and the authors' claims are only true for 1 of them. Either more primer/sets assays should be shown where the gp32/1-step is indeed as good as the 2-step, or the authors should modify the claims in the manuscript to be more honest and in line with the data.

**Response:**

We would like to thank the reviewer for pointing this out, and we understand his/her position. We however, confirmed out findings with H1N1 and the HIV gag gene, and then upon request found a strongly reduced efficacy of T4GP32 in the low copy number full HIV sample, as indicated in the first revision. We agree with Reviewer 1 that, considering we obtained this undesirable result, we should alter the manuscript to discuss this result.

**Action taken:**

After line 147, the following text was added:

“The effect of gp32 was studied using the Acrometrix HIV-1 control, and a near-negligible A weak improvement in ttp (from 29 min to 28.5 min) was found using this full length HIV-1 RNA primer set (Supplementary Information), a result that may be due to the low target concentration (100 copies/reaction). This more modest effect could be caused by the low sample concentration or by the decreased dependence of the HIV-1 gag gene primer set on the thermal annealing step. Improvements in ttp using gp32 were confirmed for Avian influenza A H5N1 RNA, decreasing ttp from 38 to 31 min in presence of gp32, compared with a ttp of 28 min for the two step process (unpublished results). “

That in circumstances where avoiding heat denaturation is paramount to NASBA utilization, then gp32 may help, but there may be sacrifices in performance depending on the assay. That is obviously not a barrier to high-throughput NASBA/TMA as the authors now even state, the Hologic and Biomerieux platforms do seem to work. Perhaps a simple point-of-care or at-home test NASBA would indeed be more practical if the 2-step protocol can be avoided, and the manuscript would be stronger if the authors present that more honestly.

**Response:**

It is unclear to the authors what changes the reviewer would like us to make, as the

*Introduction*

“ the 2-step process could be considered inconvenient for high throughput or point of care operation as each additional processing step may present an operational and engineering challenge [14]. “

*Discussion*

“A truly isothermal, single-step NASBA method is anticipated to enhance NASBA’s potential for adoption in portable diagnostic devices, as it eliminates the need for complex temperature and fluidic control, hence simplifying instrument and operational design. “

and *Conclusion*

“, removing an operational bottleneck by facilitating one-pot NASBA”

all clearly state the two step process is a bottleneck for decentralised testing, and the aim of this work it so overcome this bottleneck using a SSB.

The pandemic, and particularly Omicron wave has demonstrated that centralised PCR testing is too slow and cumbersome, and rapid antigen testing is adopted despite lower specificity, and higher false positive/false negative rates. Simplicity is the key to effective decentralised testing (see WHO ASSURED criteria), and a single step reaction is simpler than a two-step process. It does not mean the same or superior results cannot be obtained with sophisticated engineering, but if we compare it to the transport sector we have different expectations and are prepared to make different sacrifices when using a plane (complex engineering but centralised) or bicycle (less complex engineering but decentralised) for transport.

**Action taken:**

To further emphasize the focus of this work towards the field of decentralised, point of care testing, we added

“for enhanced field deployability. “ to line 91

We trust that this explanation combined with the edited text adresses the reviewers concern.

Reviewer #3: Authors of the manuscript entitled “An Improved Nucleic Acid Sequence-Based Amplification Method Mediated by T4 Gene 32 Protein” have thoroughly revised the said manuscript in the light of comments/suggestions raised by the reviewers. Responses to reviewers’ comments have satisfactorily been addressed by the authors and have also been incorporated at the appropriate places within the revised manuscript. The revised manuscript may now be accepted for publication in PLOS ONE. However, there is a couple of very minor corrections that should be done at the Journal level before publication.

**Response:**

We would like to thank this reviewer for these comments

1. Introduction section on page no. 4, line no. 73- abbreviation ‘RPA’ has been used for the first time in the manuscript, so its full form ‘Recombinase Polymerase Amplification’ should also be written at that place.

**Response:**

Thanks for pointing out this omission,

**Action taken:**

We now included the definition before using the abbreviation.

“Compared to other isothermal amplification methods, such as LAMP *or Recombinase Polymerase Amplification (RPA)*, the 2-step process could be considered inconvenient for high throughput or point of care operation as each additional processing step may present an operational and engineering challenge [14].

2. Results and Discussion section, page 5, line 97- ‘times to not include’ should be
‘times do not include’.

**Response:**

Thanks for pointing out this omission

**Action taken:**

The typographical error has been corrected and now reads “Please note the recorded amplification times do not include the extra time required for the addition of the amplification enzymes after thermal annealing using the 2-step protocol.”
