# Supplementary material for: An improved nucleic acid sequence-based amplification method mediated by T4 gene 32 protein
Source: PLoS One. 2022 Mar 24;17(3):e0265391. doi: 10.1371/journal.pone.0265391 (PMC8947125; doi:10.1371/journal.pone.0265391)
Supplement: Attachment — Submitted filename: response v3.docx [file pone.0265391.s005.docx]

We would like to thanks Reviewer 1 for clarifying her/his comments. Again, the reviewers comments are pasted in black and aligned to the right, and our response and overview of changes made to the manuscript are indicated in blue and aligned to the left of the page.

Reviewer #1: The authors have again made improvements to the manuscript and adjusted language to be in line with what is actually demonstrated by the data. So I'm mostly in agreement it can be accepted for publication, but there are still a few issues. I said in the last review that claiming the two-step nature of NASBA is the barrier to its widespread use is unfounded, that benefit would be only in an at-home or simple device...and the authors responded with
"It is unclear to the authors what changes the reviewer would like us to make". Well here's one. The very first sentence of the manuscript in the abstract is: "The uptake of Nucleic Acid Sequence-Based Amplification (NASBA) is hindered by the requirement of a thermal denaturation step to initiate the cyclic isothermal amplification." That is plainly not true. If the authors want to cite ASSURED as justification for use of a 1-step vs. 2-step test, they should consider that the E stands for "equipment-free" which doesn't really apply to a test that uses fluorescent beacons no matter how many temperatures are involved.

**Response**

We would like to thank the reviewer for her/his clarification. and now understand we failed to distinguish between the ultimate goal articulated in the WHO’s Affordable, Sensitive, Specific, User-friendly, Rapid and robust, Equipment-free and Deliverable to end-users (ASSURED) criteria, and the commonly accepted reality where of point of care diagnostic products often fail to meet part of these criteria, and are not equipment free. We specifically selected reference 1 because within its focus on nucleic acid testing, it articulates the need for

*“****Procedural simplicity***

*In order to reduce cost, prevent malfunction and achieve robust, reliable operation within a simple package an amplification technology will preferably be a “single tube” reaction with a minimal volume, employing few reagents and few fluidic manipulations.“ (Craw, LoC, 2012)*

The use of gp32 is aimed to simplify the workflow, and we acknowledge this simplification does not eliminate the need for equipment. As we now understand the standpoint of the reviewer, we amended the manuscript to prevent confusion and remove any ambiguity to the field the potential benefits of this work apply to.

**Action taken**

1. Limited the field the significance of the work applies to point of care diagnostics, and improved the articulation of the scope of the work to the simplification of the work flow by modifying the opening and closing sentence of the abstract

*The uptake of Nucleic Acid Sequence-Based Amplification (NASBA) for point of care testing may be hindered by a complexity in the workflow due the requirement of a thermal denaturation step to initiate the cyclic isothermal amplification before the addition of the amplification enzymes.*

….

*For synthetic HIV-1 RNA, gp32 was found to improve the time-to-positivity (ttp) by average of 13.6% of one-step NASBA and 6.7% of conventional NASBA for the detection of HIV-1 RNA, showing its potential for simplifying the workflow as desirable for point of care applications of NASBA.*

2. Clarifying the purpose in line 78 by adding an additional reference to [1]

*Procedural simplicity is critical reduce cost, prevent malfunction and achieve robust, reliable operation [1].*

3. And re-iterating this in the conclusion

*In conclusion, SSBs can be used to decrease the reliance on a thermal annealing step in NASBA, enhancing the procedural simplicity by facilitating one-pot NASBA.*

If the authors would simply not claim they've fixed NASBA, a method that's used worldwide every day for diagnostics, but rather keep the claims to that they've shown maybe the denaturation step could be omitted if gp32 is added then I'd be totally fine with this manuscript. The discussion and conclusion paragraph have this much better, so just change the Abstract and I say it's okay.

**Response**

Now we understand the reviewers misunderstanding of our aim of simplifying the workflow vs meeting the ASSURED criteria including being equipment free, we amended the manuscript as indicated above to clarify the significance of this work is limited to the workflow, and will not allow NASBA to meet he ASSURED criteria. In response to the reviewers wish to amend the abstract, we provide the amended abstract below.

**Action taken:**

Amended abstract (new text in red)

The uptake of Nucleic Acid Sequence-Based Amplification (NASBA) for point of care testing may be hindered by a complexity in the workflow due the requirement of a thermal denaturation step to initiate the cyclic isothermal amplification before the addition of the amplification enzymes. Despite reports of successful enhancement of other DNA and RNA amplification methods using DNA and RNA binding proteins, this has not been reported for NASBA. Here, three single-stranded binding proteins, RecA, Extreme Thermostable Single-stranded binding protein (ET SSB) and T4 gene gp32 protein (gp32), were incorporated in NASBA protocol and used for single pot, one-step NASBA at 41 °C. Indeed, all SSBs showed significantly improved amplifications compared with the 2-step process, but only gp32 showed no non-specific aberrant amplification, and slightly improved the time-to-positivity in comparison with the conventional NASBA. For synthetic HIV-1 RNA, gp32 was found to improve the time-to-positivity (ttp) by average of 13.6% of one-step NASBA and 6.7% of conventional NASBA for the detection of HIV-1 RNA, showing its potential for simplifying the workflow as desirable for point of care applications of NASBA.
